# Bronchial Casts and Pandemic (H1N1) 2009 Virus Infection

**DOI:** 10.3201/eid1602.091607

**Published:** 2010-02

**Authors:** Maki Hasegawa, Yasuji Inamo, Tatsuo Fuchigami, Koji Hashimoto, Miyuki Morozumi, Kimiko Ubukata, Haruo Watanabe, Takashi Takahashi

**Affiliations:** Nihon University Nerima-Hikarigaoka Hospital, Tokyo, Japan (M. Hasegawa, Y. Inamo, T. Fuchigami, K. Hashimoto); Graduate School of Infection Control Sciences, Kitasato University, Tokyo (M. Morozumi, K. Ubukata, T. Takahashi); National Institute of Infectious Diseases, Tokyo (H. Watanabe)

**Keywords:** Bronchial casts, plastic bronchitis, atelectasis, influenza, H1N1, pandemic, virus, pediatric patients, expedite, letter

**To the Editor:** In the late 1990s, triple-reassortant influenza A viruses containing genes from avian, human, and swine influenza viruses emerged and became enzootic in swine herds in North America (*1*). The first 11 human cases of novel influenza A virus infection were reported to the Centers for Disease Control and Prevention (CDC; Atlanta, GA, USA) from December 2005 through February 2009 (*1*). In response to those reports, surveillance for human infection with nonsubtypeable influenza A viruses was implemented.

In the spring of 2009, outbreaks of febrile respiratory infections caused by a novel influenza A virus (H1N1) were reported among persons in Mexico, the United States, and Canada (*2*). Patient specimens were sent to CDC for real-time reverse transcription–PCR (RT-PCR) testing, and from April 15 through May 5, 2009, a total of 642 infections with the virus, now called pandemic (H1N1) 2009 virus, were confirmed. Of those 642 patients, 60% were <18 years of age, indicating that children may be particularly susceptible to pandemic (H1N1) 2009 (*2*).

Children and adults with preexisting underlying respiratory conditions, such as asthma, are at increased risk for complications from infection with pandemic (H1N1) 2009 virus. One possible complication is plastic bronchitis, a rare respiratory illness characterized by formation of large gelatinous or rigid branching airway casts (*3*). Plastic bronchitis is a potentially fatal condition induced by bronchial obstruction from mucus accumulation resulting from infection, inflammation, or vascular stasis (*4*). We report a case of bronchial casts that caused atelectasis of the right lung of a child infected with influenza A pandemic (H1N1) 2009 virus.

A 6-year-old boy with asthma and a 1-day history of fever and cough was referred to a hospital pediatrics department because of dyspnea. Clinical examination at hospital admission found respiratory distress, as shown by tachypnea (respiratory rate 66 breaths/min) and inspiratory retraction, deficient vesicular sounds over the right lung field, elevated blood levels of immunoglobulin E (1,770 IU/mL) and a reduced number of lymphocytes (483 cells/μL), and radiographic evidence of atelectasis of the right lung and hyperinflation of the left lung without air leakage (Figure, panel A). Pandemic (H1N1) 2009 virus infection was confirmed by real-time RT-PCR, as described (*5*), of an endotracheal aspirate. Real-time PCR ruled out *Streptococcus pneumoniae, Haemophilus influenzae, Mycoplasma pneumoniae, Legionella pneumophila, Chlamydophila pneumoniae, S. pyogenes*, respiratory syncytial viruses A and B, seasonal influenza viruses A and B, parainfluenza viruses 1–3, rhinovirus, enterovirus, human metapneumovirus, human bocavirus, and adenovirus (*6*). While the patient was breathing room air, his percutaneously monitored oxygen saturation was 86%; respiratory support by mechanical ventilation was then initiated. Mucus casts were extracted by intratracheal suction (Figure, panel B). The patient was treated with an inhaled bronchodilator, intravenous methylprednisolone (20–60 mg/day for 7 days), and antiviral (oseltamivir) and antimicrobial (ampicillin/sulbactam) drugs.

**Figure F1:**
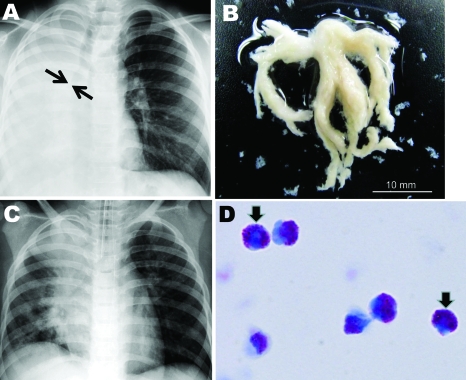
A) Chest radiograph obtained at hospital admission from a child infected with influenza subtype H1N1 virus. The image shows atelectasis of the right lung and hyperinflation of the left lung; arrows indicate obstruction of the right main bronchus. B) Macroscopic bronchial casts extracted by intratracheal suction. C) Chest radiograph obtained on hospital day 2, indicating partial resolution of atelectasis of the right lower lobe. D) Light micrograph of casts, characterized by predominant eosinophil infiltration (>90% of cells) (May-Giemsa stain, original magnification ×1,000). Arrows indicate typical eosinophil granules.

On hospital day 2, chest radiographs showed that atelectasis of the right lower lobe had partially resolved (Figure, panel C). A histologic examination of casts (May**-**Giemsa stain; Figure, [Fig F1]panel D) indicated a mucoid substance containing a predominantly eosinophilic infiltrate (>90% of cells). The patient’s respiratory condition during 11 days of oxygen supplementation gradually improved, and he was discharged on hospital day 18.

Plastic bronchitis is related mainly to respiratory, cyanotic cardiac (post-Fontan), and hematologic (sickle cell anemia) diseases. A diagnosis of plastic bronchitis is determined on the basis of clinical findings (pointing to allergic and asthmatic, cardiac, or idiopathic etiologies) and pathologic findings (inflammatory vs. noninflammatory) on examination of casts (*3*). Inflammatory casts contain fibrin, eosinophils, and Charcot-Leyden crystals; noninflammatory casts contain mucin and exhibit vascular hydrostatic changes. The case presented here was the allergic-inflammatory type of plastic bronchitis.

Various treatments for plastic bronchitis have been described and vary from cast removal by expectoration or by bronchoscopy (*7*,*8*). Other interventions involve cast disruption by tissue plasminogen activator or urokinase and prevention of cast formation by use of mucolytic agents, steroids, or anticoagulants. However, evidence remains anecdotal because too few plastic bronchitis patients are available for clinical trials. Details of steroid dosage will need to be clarified for pandemic (H1N1) 2009 virus–infected children with respiratory distress from bronchitis and pneumonia.

In Iran during 1998–2001, avian influenza (H9N2) infection among broiler chickens resulted in 20%–60% mortality rates on affected farms (*9*). Macroscopic examination of specimens from infected chickens showed extensive hyperemia of the respiratory tract, followed by exudate and casts extending from the tracheal bifurcation to the secondary bronchi. Light microscopy indicated severe necrotizing tracheitis. Pandemic (H1N1) 2009 can produce similar airway cast formation in humans; severe respiratory distress reflects extensive obstruction of the respiratory system.

Healthcare providers should be aware of the possibility of bronchial casts when examining children with influenza (H1N1) infection accompanied by atelectasis. Steroids can be administered early in infection to avoid cast formation, and antiviral drug therapy and respiratory support can be used for influenza (H1N1)–infected children in whom airway casts have developed.
